# Reduced heart rate variability is related to the number of metabolic syndrome components and manifest diabetes in the sixth Tromsø study 2007–2008

**DOI:** 10.1038/s41598-022-15824-0

**Published:** 2022-07-14

**Authors:** Naomi Azulay, Roy Bjørkholt Olsen, Christopher Sivert Nielsen, Audun Stubhaug, Trond Geir Jenssen, Henrik Schirmer, Arnoldo Frigessi, Leiv Arne Rosseland, Christian Tronstad

**Affiliations:** 1 Department of Research and Development, Division of Emergencies and Critical Care, Oslo University Hospital, Oslo, Norway; 2 Institute of Clinical Medicine, University of Oslo, Oslo, Norway; 3 Department of Anesthesiology and Intensive Care, Sørlandet Hospital, Arendal, Norway; 4 Department of Chronic Diseases, Norwegian Institute of Public Health, Oslo, Norway; 5 Department of Pain Management and Research, Oslo University Hospital, Oslo, Norway; 6 Department of Transplantation Medicine, Section of Nephrology, Oslo University Hospital, Rikshospitalet, Oslo, Norway; 7 Metabolic and Renal Research Group, Faculty of Health Sciences, UiT- The Arctic University of Norway, Tromsø, Norway; 8 Department of Cardiology, Akershus University Hospital, Lørenskog, Norway; 9 Oslo Centre for Biostatistics and Epidemiology, University of Oslo, Oslo, Norway; 10 Oslo Centre for Biostatistics and Epidemiology, Oslo University Hospital, Oslo, Norway; 11 Department of Clinical and Biomedical Engineering, Oslo University Hospital, Oslo, Norway

**Keywords:** Cardiovascular diseases, Diabetes, Metabolic syndrome

## Abstract

Both diabetes mellitus (DM) and the metabolic syndrome (MetS) are associated with autonomic neuropathy, which predisposes to cardiac events and death. Measures of heart rate variability (HRV) can be used to monitor the activity of the autonomic nervous system (ANS), and there are strong indications that HRV can be used to study the progression of ANS-related diabetes complications. This study aims to investigate differences in HRV in healthy, MetS and diabetic populations. Based on 7880 participants from the sixth health survey in Tromsø (Tromsø 6, 2007–2008), we found a significant negative association between the number of MetS components and HRV as estimated from short-term pulse wave signals (PRV). This decrease in PRV did not appear to be linear, instead it leveled off after the third component, with no significant difference in PRV between the MetS and DM populations. There was a significant negative association between HbA1c and PRV, showing a decrease in PRV occurring already within the normal HbA1c range. The MetS and DM populations are different from healthy controls with respect to PRV, indicating impaired ANS in both conditions. In the future, a study with assessment of PRV measurements in relation to prospective cardiovascular events seems justified.

## Introduction

Diabetes mellitus was by 2017 the fourth leading cause of disability worldwide^1^ and is rapidly becoming more prevalent also in the developing world^2^. Metabolic syndrome (MetS) is a disorder defined by the elevated risk of developing cardiovascular disease (CVD) and diabetes mellitus type 2 (DMT2)^3^ and is measured through a cluster of risk factors, or components, for these diseases. Which specific components to include in the cluster and where to set the cut-off values have been revised over time, as has the algorithm used to define the MetS. In this study, the harmonized definition, agreed upon by the International Diabetes Federation and the American Heart Association/National Heart, Lung, and Blood Institute in 2009, is used^3^. This definition states that at least three out of the following five criteria must be met for a diagnosis to be made: (1) abdominal obesity, (2) elevated levels of triglycerides, (3) reduced HDL cholesterol, (4) elevated blood pressure, and (5) elevated fasting glucose. The high blood pressure and low HDL cholesterol criteria have sex-specific cut-off values, and the central obesity criterion has both sex and ethnicity-specific cut-offs. In MetS, the components occur together more often than chance alone would indicate^4^.

Measurement of heart rate variability (HRV) is a well-known method for studying activity in the autonomic nervous system. Previous studies have found altered (mostly reduced) HRV in the MetS population, and an association between each of the components of MetS and HRV^5^. This indicates that the MetS and its components adversely affect cardiac autonomic control, which in turn may contribute to the increased risk of CVD observed in the MetS and DMT2 populations^6^. CVD is a well-known comorbidity of diabetes, and the MetS is by definition also associated with a higher risk of CVD.

Cardiac autonomic neuropathy (CAN) is a serious complication of diabetes that results from damage to the nerves that innervate the heart. It is an important predictor of cardiovascular events in the diabetic population^7^ and is associated with an increased risk of all-cause and cardiovascular mortality^8,9^. There is increasing evidence that CAN is also present in prediabetes and MetS^10^, suggesting that the effects on the nervous system can occur before the development of DMT2. A 2015 study found that there was an increase in the prevalence of CAN (defined as ≥ 2 abnormal HRV indices out of 4 possible) across abnormal glucose levels (impaired glucose tolerance (IGT), impaired fasting glucose (IFG), both IGT and IFG, newly diagnosed diabetes and known diabetes)^11^.

Previous studies have found evidence for an association between HRV and the number of metabolic syndrome components, with number of participants ranging from 35 to 1889^5,12–14^. None had diabetes as a separate group of comparison.

HRV is by standard calculated from the electrocardiogram (ECG), where the heartbeat intervals are accurately determined based on the distances between R peaks. Recently, estimates of HRV based on pulse wave measurements (pulse rate variability, PRV) have gained increased interest due to the availability of photoplethysmographic sensors in wearable devices. However, the accuracy of these estimates has only been proven to be sufficiently accurate for healthy, mostly younger, subjects at rest^15^. Both mental stress and, especially, physical activity, can result in lower accuracy between HRV and PRV^16^.

In addition to the signal modality (ECG or photoplethysmography), estimates of HRV may also depend on the length of the recording that is used for calculation, as longer periods will contain more slow-varying heart rate changes and has a higher chance of capturing the typical HRV of the subject compared to short-term (~ 5 min) or ultra-short-term (down to 10 s) recordings. Due to the time cost of long or short-term HRV assessment as a barrier for integration in routine medical practice, ultra-short-term HRV estimates are of interest^17^. Several recent studies have evaluated the reliability of ultra-short-term HRV compared to HRV derived from longer recordings^17–21^, where minimal recording lengths have been suggested ranging from 20 to 300 s depending on the type of HRV metric.

HRV can be represented both by time-domain and frequency-domain measures. In this study, we will use the two simplest and most commonly used time-domain measures calculated from ultra-short-term recordings: SDNN and RMSSD^22^. SDNN is the standard deviation of the time between beats and represents the total variation in heart rate, while RMSSD stands for root mean square of successive differences and represents variation between successive beats.

This study is based on ultra-short PRV recordings taken in connection with a cold pressure test (CPT) at the Tromsø study. SDNN and RMSSD are calculated from the periods before (~ 30 s) and after (~ 60 s) the CPT.

We hypothesized that the altered HRV seen in MetS is related to the number of metabolic syndrome components (MetS levels) present, and that the strongest alteration is in the people with manifest diabetes. In either case, we also tested whether certain MetS components or certain combinations of components show stronger association with HRV than others.

In addition, we have assessed the relationship between HRV and HbA1c (glycated hemoglobin) in the sample, and due to the relationship between albumin-creatinine ratio (ACR) and microvascular disease, a possible relationship between ACR and HRV has also been studied. Lastly, we investigated if the relationship between HRV and the number of MetS components was similar among those with and without known CVD.

## Results

### Descriptive statistics

7704 participants had readable PRV data from the pre-CPT period. 496 of them had diabetes, with imputations it ranged from 496 to 506. 2360 had MetS (imputation range 2485–2505). 634 had CVD (imputation range 642–649). 4327 had data on albuminuria.

**Figure 1:**
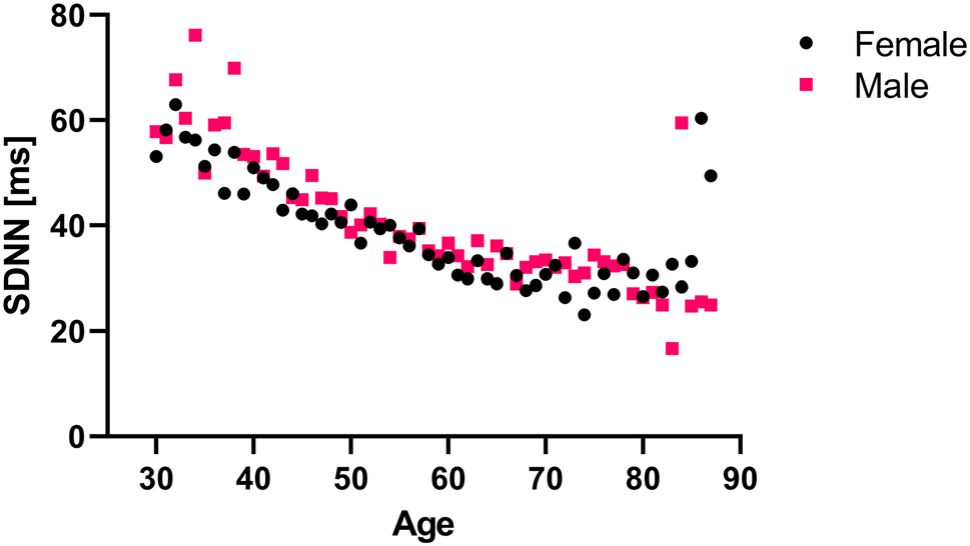
Mean SDNN (ms) as a function of age for men and women from the period before the cold pressure test.

Figure 1 shows mean SDNN from the pre-CPT period as a function of age in the sample, grouped by sex. It shows a negative trend for age, and higher PRV for men than women. Both relationships were significant for the SDNN outcomes (age: p < 0.001 pre-CPT, p < 0.001 post-CPT, sex: p < 0.001 pre-CPT, p = 0.020 post-CPT), and all models were consequently adjusted for sex and age (centered at 55 years). Table 1 shows characteristics for healthy controls, MetS and DM subgroups, respectively (all variables used in the study are listed in Table 2).

**Table 1:** Mean and standard deviation (continuous variables) or the percentage and number of participants (categorical variables) of select population characteristics in the control, MetS and DM subgroups.

Variable	Control (n = 7305)	MetS (n = 3909)	Diabetes Mellitus (n = 964)	P-value
Age (SD)	54.9 (12.3)	60.5 (11.9)	64.2 (11.3)	< 0.001
Sex % female (n)	57.2 (4178)	47.1 (2066)	48.3 (466)	< 0.001
Hypertension % (n)	46.6 (3401)	86.4 (3377)	87.2 (834)	< 0.001
High triglycerides % (n)	9.6 (702)	82.4 (3220)	70.0 (657)	< 0.001
High glucose % (n)	5.6 (409)	32.7 (1278)	70.5 (672)	< 0.001
Central obesity % (n)	66.5 (4858)	93.4 (3652)	88.7 (813)	< 0.001
Low HDL % (n)	4.6 (337)	59.0 (2306)	61.3 (571)	< 0.001
SDNN pre-CPT ms (SD)	42.7 (27.7)	36.3 (28.7)	33.7 (31.2)	< 0.001
SDNN post CPT ms (SD)	62.2 (36.0)	53.2 (35.6)	49.4 (34.9)	< 0.001
RMSSD pre-CPT ms (SD)	33.2 (26.6)	29.5 (30.3)	29.6 (35.2)	< 0.001
RMSSD post CPT ms (SD)	37.8 (30.0)	33.4 (32.5)	32.4 (36.2)	< 0.001

P-values from ANOVA (continuous variables) or chi-square tests (categorical variables).

**Table 2:** Characteristics of the participants (n = 12 981), with mean and standard deviation (continuous variables), the percentage and number of participants (categorical variables), or median and interquartile range (IQR; very skewed distributions). Column-wise percentage missing, data source and the variable's role in the analysis are also listed.

Variable	Mean (SD), median (IQR) or percentage (n)	% missing	Source	Type
Age year (SD)	57.5 (12.7)	0.0	Questionnaire	Covariate
Sex % female (n)	53.4 (6928)	0.0	Questionnaire	Covariate
Waist cm (SD)	94.9 (12.2)	3.6	Clinical examination	Explanatory
Self-reported diabetes % (n)	5.0 (634)	2.1	Questionnaire	Explanatory
Age diabetes diagnosed year (SD)	53.8 (15.3)	26.8	Questionnaire	Exclusion
HbA1c mmol/mol (SD)	38.2 (7.1)	1.6	Blood sample	Explanatory
Triglycerides mmol/l (SD)	1.5 (1.0)	1.2	Blood sample	Explanatory
High density lipoprotein cholesterol mmol/l (SD)	1.5 (0.4)	1.2	Blood sample	Explanatory
Glucose mmol/l (SD)	5.2 (1.2)	1.2	Blood sample	Explanatory
Time since last meal hours (SD)	3.2 (1.9)	1.0	Questionnaire	Explanatory
Systolic blood pressure mmHg (SD)	135.6 (23.0)	0.6	Clinical examination	Explanatory
Diastolic blood pressure mmHg (SD)	77.8 (10.7)	0.6	Clinical examination	Explanatory
Use of lipid lowering drugs % (n)	14.6 (1866)	1.7	Questionnaire	Explanatory
Antihypertensive medication % (n)	22.6 (2891)	1.4	Questionnaire	Explanatory
Use of insulin % (n)	1.7 (211)	2.7	Questionnaire	Explanatory
Antidiabetic tablets % (n)	3.4 (437)	2.1	Questionnaire	Explanatory
Pregnant % (n)	0.4 (28)	7.6	Questionnaire	Exclusion
Heart attack % (n)	5.4 (682)	2.1	Questionnaire	Explanatory
Angina % (n)	5.0 (628)	2.4	Questionnaire	Explanatory
Stroke % (n)	2.9 (362)	2.4	Questionnaire	Explanatory
Atrial fibrillation % (n)	6.0 (754)	3.5	Questionnaire	Exclusion
Albumin-creatinine ratio mg/mmol (IQR)	0.4 (0.6)	5.6	Urine sample	Explanatory
SDNN pre-CPT ms (SD)	40.0 (28.6)	1.8	CPT	Outcome
SDNN post CPT ms (SD)	58.5 (36.2)	6.8	CPT	Outcome
RMSSD pre-CPT ms (SD)	31.9 (28.8)	1.8	CPT	Outcome
RMSSD post CPT ms (SD)	36.2 (31.8)	6.8	CPT	Outcome

Where relevant, data that is missing by design is excluded from the calculation of the missing percentage. All numbers are from before exclusions. CPT cold pressure test.

**Figure 2:**
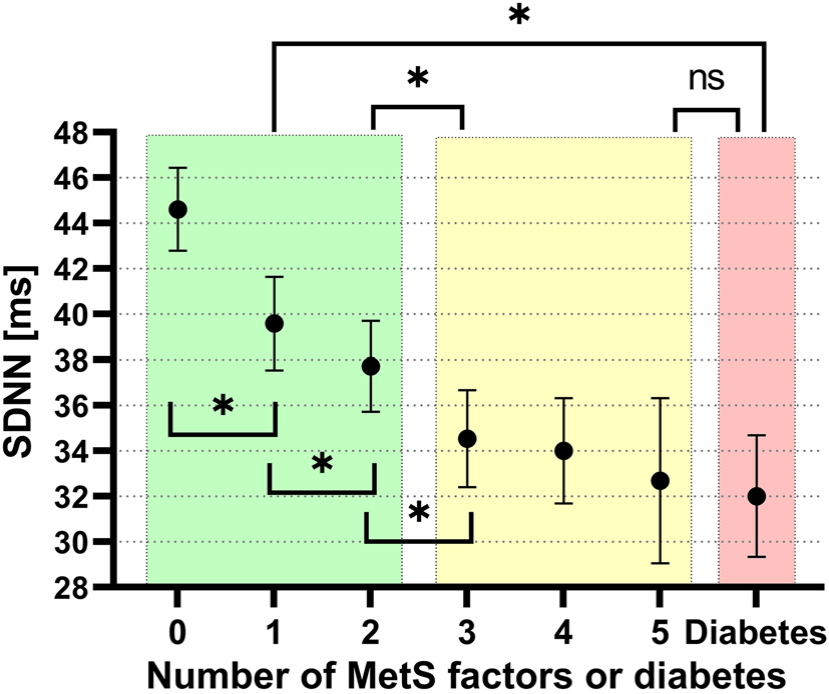
Mean and 95% confidence intervals of SDNN from the pre-CPT period for participants having different numbers of metabolic syndrome components or diabetes. Green area represents healthy subjects and yellow area represents subjects with metabolic syndrome according to current definitions. *p < 0.05 from contrast analysis and Tukey’s tests. Only Tukey tests from adjacent groups are shown in this plot, for a complete overview, see supplementary figure S3.

The Pearson correlation between measurements taken pre and post CPT was 0.51 for SDNN and 0.66 for RMSSD. The correlation between baseline heart rate and pre-CPT SDNN was -0.29. In the presentation of HRV versus number of MetS components, all Figs. (2, 4, S1, S3, S5, S6) show the effect estimates (with their confidence intervals) added to the intercept estimate. After the centering, the intercept represents the HRV levels for women at age 55 with no MetS components.

### Alteration in HRV increases with increasing number of metabolic syndrome components, with the strongest alteration in manifest diabetes

Mean SDNN from the pre-CPT period (adjusted for sex and age) was 44.6, 39.6, 37.7, 34.5, 34.0, 32.7 for increasing number of MetS components, and 32.0 for manifest diabetes (Fig. 2). The MetS level variable contributed significantly to the ANCOVA model (p < 0.001), and all levels of MetS had significantly reduced SDNN compared to participants having no MetS components fulfilled (p < 0.001 for all comparisons). The effect of sex and age was 2.7 ms and -0.5 ms per year, respectively. The model explained about 12% of the SDNN variance (R^2^ = 0.118). As shown in Fig. 2, contrast analysis showed that the MetS group (3–5 components fulfilled) differed significantly from the healthy group (0–2 components; p < 0.001), and the same was found for participants with diabetes vs healthy persons (p < 0.001). The difference between the MetS and diabetes groups was not statistically significant (p = 0.200). Pairwise comparisons with Tukey’s method showed that SDNN for 0, 1 and 2 components were significantly different compared to all other groups. Having 3 components was significantly different from diabetes (p = 0.038). There were no other differences between the groups with 3–5 MetS levels and diabetes, consistent with a non-linear leveling off effect. In other words, there is a significant reduction in SDNN for every added MetS component up to three components, but not beyond that point. Results were similar for the other outcome variables, as shown in supplementary figures S1 and S2. The proportion of explained PRV was lower when RMSSD was used as outcome, at a level of 4–5%. The PRV was higher for SDNN outcomes compared to RMSSD, and in the post-CPT period as compared to pre-CPT (see supplementary figure S1). This analysis was repeated with participants on beta blockers excluded, see supplementary figure S3 for results. A breakdown of the percentage of fulfilled specific MetS components within each MetS level is shown in Table 3.

**Table 3:** Overview of the percentage of participants with a given number of MetS components or diabetes that fulfills each of the specific components. E.g., 75% of participants with two of the MetS components have hypertension as one of them.

	Number of metabolic syndrome components				Diabetes	All
	1	2	3	4		
Hypertension	26.9	75.0	77.1	95.3	88.5	61.8
High triglycerides	4.9	17.0	69.9	97.5	69.0	37.4
High glucose	3.1	9.0	28.0	26.0	100	13.8
Central obesity	64.1	90.7	90.4	97.8	89.9	78.0
Low HDL	1.0	8.4	34.6	83.4	59.2	25.4

In the models with the specific components and diabetes as dichotomized variables, all components contributed significantly to the alteration in PRV when SDNN from the post-CPT was used as outcome. For pre-CPT SDNN, low HDL was not a significant component. For both RMSSD outcomes, diabetes and central obesity were not significant. In the models where low HDL was significant, it was associated with a higher PRV value. The estimates, 95% confidence intervals and p-values from all four models can be found in Table 4.

**Table 4:** Estimate (95% confidence interval) and p-value of each MetS component for every PRV outcome. The estimates are the mean change in PRV when specific MetS components are acquired, when the other variables are kept constant.

	Outcome variable			
	Pre CPT SDNN	Post CPT SDNN	Pre CPT RMSSD	Post CPT RMSSD
Hypertension	− 3.0 (− 4.2, − 1.9) *	− 6.2 (− 7.7, − 4.6) *	− 3.0 (− 4.2, − 1.9) *	− 4.9 (− 6.2, − 3.6) *
Diabetes	− 3.5 (− 5.6, − 1.5) *	− 4.5 (− 7.5, − 1.6) *	− 2.0 (− 4.0, 0.1)	− 2.1 (− 4.5, 0.4)
High triglycerides	− 3.5 (− 4.8, − 2.3) *	− 4.3 (− 6.1, − 2.6) *	− 3.9 (− 5.1, − 2.6) *	− 3.5 (− 5.0, − 2.1) *
High glucose	− 2.4 (− 3.9, − 1.0) *	− 4.0 (− 6.0, − 2.0) *	− 2.5 (− 3.9, − 1.0) *	− 2.5 (− 4.1, − 0.8) *
Central obesity	− 2.3 (− 3.5, − 1.0) *	− 3.5 (− 5.3, − 1.8) *	− 0.3 (− 1.6, 0.9)	− 0.8 (− 2.2, 0.7)
Low HDL	− 0.4 (− 1.8, 1.0)	2.0 (0.1, 3.9) *	1.5 (0.7, 2.9) *	2.3 (0.7, 3.9) *

MetS metabolic syndrome, CPT cold pressure test. * p < 0.05.

There was a significant interaction between central obesity and blood pressure in the pre-CPT SDNN model (p = 0.032), but not with any of the other outcomes. No three-way interactions were statistically significant for any outcome.

### Alteration in HRV increases with increasing HbA1c level

**Figure 3:**
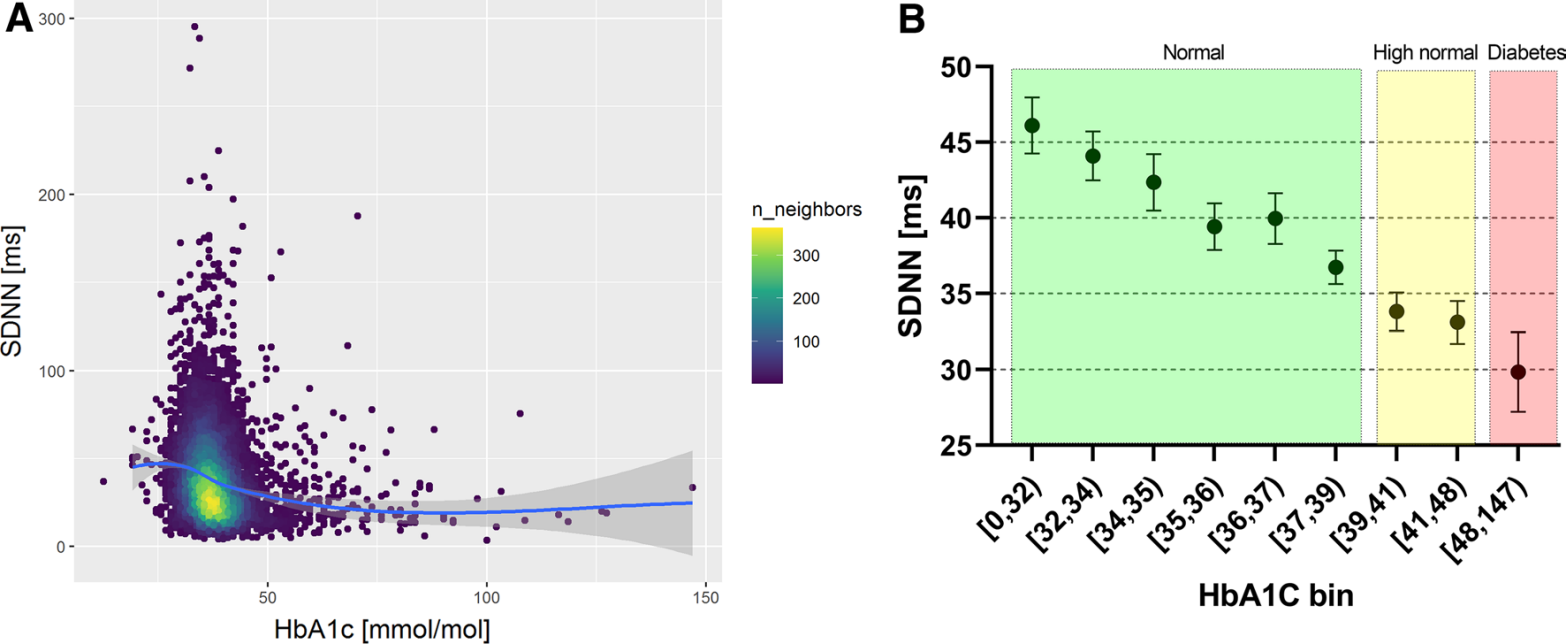
**A** Point density plot for the association between pre-CPT SDNN and HbA1c. A lighter color means that there is a higher density of observations in that area. The solid line is estimated with Locally Estimated Scatterplot Smoothing (LOESS), representing the smooth relationship between the variables (the shaded error band represents the 95% confidence interval of the LOESS). **B**. Mean and 95% confidence intervals of SDNN pre-CPT for participants grouped according to different HbA1c ranges. The ranges are selected in order to keep group sizes similar up to the defined level for diabetes at 48 mmol/mol and above. An HbA1c value between 39 and 48 mmol/mol is considered high.

The relation between pre-CPT SDNN and HbA1c is presented in Fig. 3, both as a point density plot (Fig. 3A) and as the mean SDNN categorized in intervals of HbA1c for a clearer representation of the association (Fig. 3B). These unadjusted data revealed a substantial decrease in PRV with increasing levels of HbA1c up to the defined range for diabetes (≥ 48 mmol/mol).

Both linear regression (p < 0.001) and GAM modeling (p < 0.001) showed a significant association between HbA1c and pre-CPT SDNN (R^2^ = 11% for both). Although HbA1c was included as a non-linear term in the GAM, the prediction line had a linear shape.

The results were similar for the other outcomes, but the R^2^ for the RMSSD outcomes were lower, 4–5% for both regression and GAM. The statistical effect of HbA1c was larger in the post-CPT models, and the GAM’s prediction lines had slightly more non-linear shapes.

An additional test with HbA1c as a categorical explanatory variable according to clinical cut-offs (normal: < 39 mmol/mol, high normal: 39–48 mmol/mol, DM: ≥ 48 mmol/mol) showed that HbA1c levels, either in the high normal (prediabetes) or diabetes ranges, had in either case significantly lower SDNN pre-CPT than the normal group (p < 0.001).

### HRV is associated with albuminuria independently of HbA1c

A linear regression model with log transformed ACR showed a significant negative relationship for the SDNN outcomes (p = 0.037 pre-CPT, p = 0.003 post-CPT, R^2^ = 3% both models). Log ACR was not significantly associated with RMSSD (p = 0.693 pre-CPT, p = 0.924 post-CPT). When adding HbA1c to the SDNN models, the log ACR estimate remained negative, but only with statistical significance in the post-CPT period (p = 0.184 pre-CPT, p = 0.020 post-CPT). HbA1c remained significant in all models (p < 0.001 pre-CPT SDNN, p < 0.001 post-CPT SDNN, p = 0.001 pre-CPT RMSSD, p = 0.002 post-CPT RMSSD). The interaction between HbA1c and log ACR was significant for all outcomes except pre-CPT SDNN (p = 0.142 pre-CPT SDNN, p = 0.002 post-CPT SDNN, p = 0.043 pre-CPT RMSSD, p < 0.001 post-CPT RMSSD).

The untransformed association between pre-CPT SDNN and different ranges of HbA1c with and without moderately or severely increased albuminuria (ACR above 3.4 mg/mmol) is presented in supplementary figure S4.

### Alteration in HRV increases with increasing number of metabolic syndrome components, with the strongest alteration in manifest diabetes in subjects with and without known CVD.

**Figure 4:**
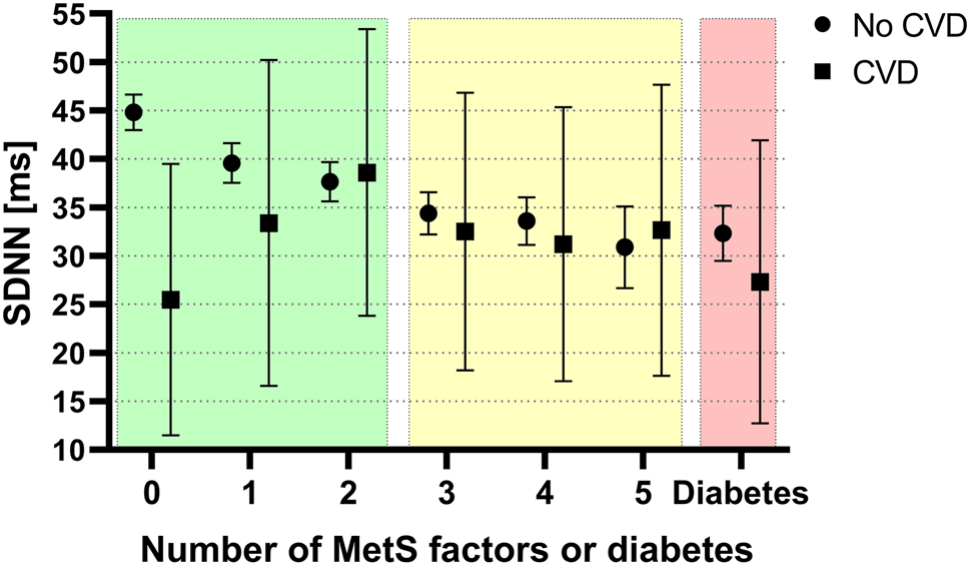
Mean and 95% confidence intervals of SDNN from the pre-CPT period for participants having different numbers of metabolic syndrome components or diabetes, stratified on CVD status. Green area represents healthy subjects and yellow area represents subjects with metabolic syndrome according to current definitions. Only 636 participants had known CVD, resulting in wider confidence intervals compared to the group without CVD.

The analyses of the main hypothesis were repeated, stratified by CVD status. The healthy group had similar results as in the main results, with the same contrasts being significant, and similar results for the Tukey tests (except for the pairwise difference between 3 components and diabetes which was no longer significant). The group with CVD, consisting of 634 participants, did not seem to follow the same pattern as the healthy group. The same contrast analysis was performed, but no contrasts were significant with any of the outcomes. The Tukey tests only showed that the level with 2 components was significantly different from most of the other levels. The result for the pre-CPT SDNN outcome is presented in Fig. 4. This analysis was repeated with participants on beta blockers excluded, see supplementary figure S6 for results.

The models were also run on unimputed data, indicating that imputation did not have a large effect on the overall conclusions (results not presented here, but available on request).

## Discussion

The results show that PRV was significantly reduced in subjects having one or more metabolic syndrome components or diabetes compared to healthy subjects. We found a significant decrease in PRV with increasing HbA1c up to the defined range for diabetes. The results regarding albuminuria were ambiguous. The pattern from the main results was not present in the group with known CVD. Results were generally stronger with SDNN as the outcome as compared to RMSSD.

The decrease in PRV with increasing levels of MetS was non-linear and plateaued after the third component. This leveling at a low PRV for three MetS levels or more is of interest to the definition of the metabolic syndrome and support the ATP III definition^23^, where a diagnosis of metabolic syndrome can be made when three of these components are present.

We expected to find a further reduction in PRV going from five levels to the diabetes group, but these groups had equal mean PRV. One explanation could be that the prevalence of known CVD in this dataset is higher in the group with all MetS components present than in those with diabetes, 32% versus 19%. The age distributions in the two groups are similar. In addition, some of the diabetes patients are well controlled, e.g., 23% of them have HbA1c levels below the cut-off for diabetes. Virtually all subjects with diabetes in this dataset have one or more component of MetS and 85% have MetS (three or more components, when counting the glucose criterion).

As shown in supplementary Fig. S2, the PRV was higher during the post-CPT period compared to pre-CPT. This could be explained as an effect of the CPT, as the test induces a significant increase in blood pressure and a sympathetic activation, with a possible parasympathetic compensation after the test. In addition, the longer recording period in the post-CPT phase could include more slow-varying heart rate patterns and a possible effect of withdrawing the hand from the cold water, increasing the HRV estimate in particular for the SDNN variable.

The components that were most influential on PRV differed depending on the outcome. Many earlier studies have presented either the correlation between MetS components and HRV (based on ECG recording), or the coefficient values from multiple regression using the continuous variables behind the MetS components. The strongest component (with highest correlation or the largest regression coefficient) is inconsistent across studies, sex, analysis method and type of HRV variable, and is therefore not easily comparable to this study. In previous studies, participants with diabetes are also generally either excluded completely or included in groups of MetS, as opposed to this study where they are presented as a separate group.

With respect to frequency domain HRV, every component has in some way been reported as the most influential, depending on the study and method of analysis. For time domain measures, HDL is the only component consistently not listed among the most influential predictors according to our knowledge. Some studies have found this as the weakest contributor^24–26^, especially for SDNN. In one study HDL was the second most influential for SDNN, but also the only component not significant for RMSSD^12^. All these four studies were based on correlations. In two studies that reported regression coefficients for SDNN and RMSSD, one study found that systolic blood pressure was the only component that was significant for both measures^27^. The other study did not find any significance for RMSSD, but triglycerides and systolic blood pressure were significant for SDNN, with triglycerides having the strongest association. Two studies used dichotomized MetS components in accordance with this study, but none of those are directly comparable as both used frequency domain measures^28,29^. All significant associations between HRV and HDL in the mentioned studies were positive, while our study showed an inverse relation.

The interaction between central obesity and blood pressure was only significant in pre-CPT SDNN, and therefore we do not consider it to be a rigorous finding. No other two-way or any three-way interactions were detected given the statistical power of the sample. We did not find studies looking for interactions among the components in a comparable way.

A 2014 systematic review by Stuckey et al.^5^ of the literature on the association between the number of metabolic syndrome components and heart rate variability between 1999 and 2012, concluded that for SDNN, there is convincing evidence of reduction in HRV with increasing number of metabolic syndrome components. Two studies tested the difference in SDNN between 0 and 1 components, one of them found a significant difference. Compared to our study, they all had less data (< 2000 participants), none used the harmonized definition of MetS, and only one used RMSSD as an HRV measure. Many grouped several MetS levels together. We used different analysis methods to answer more specific questions related to MetS, diabetes and CVD. In addition, our study was an opportunity to see if the association holds with ultra-short PRV data as a surrogate for longer ECG-based HRV recordings, which was confirmed. Since the review by Stucky et al., we could find three more papers addressing this topic. Bhagyashree et al.^14^ grouped the components and found differences between 0–2 and 4–5 components and between 3 and 4–5 components despite having only 90 participants. There were no differences between 0–2 and 3 components. Yoo et al.^12^ had more participants (N = 1200), but also grouped the number of components and found statistically significant differences between all groups, using Tukey test. Véber et al.^13^ used number of MetS components as a covariate in a regression model and found a significant relationship to SDNN based on 35 middle aged men. All nine studies found on this topic used ECG recordings, in contrast to the plethysmography-derived PRV used in our study.

Figure 3A shows a relationship between SDNN and HbA1c that is visually not linear, but binning of HbA1c (Fig. 3B) into intervals of different ranges reveals a clear monotonous negative trend. The GAMs also reported nearly linear relations, especially in the normal HbA1c range, even though it was included as a non-linear term. From Fig. 3B we see that the reduction in SDNN starts at an early level of HbA1c and gets progressively lower with increasing HbA1c. This happens well below the HbA1c cut-off value for DMT2 (48 mmol/mol), beginning already in the normal range (< 39 mmol/mol). According to a meta-analysis by Hillebrand et al.^30^, an increase in SDNN of 1% results in approximately 1% lower risk of a first incident of CVD. With this assumption, the decrease in average SDNN seen in Fig. 3B within the normal HbA1c range could translate to an increase in CVD risk of about 20%. This is consistent with data from the EPIC-Norfolk study^31^ that showed a positive continuous relationship between HbA1c concentration and rates of CVD in men, starting well within normal ranges of HbA1c. Among people with diabetes, data from the UKPDS 35^32^ shows that there is no definite lower HbA1c threshold for microvascular or macrovascular events, although the event rate is much lower with lower HbA1c levels (Stratton et al. BMJ 2000).

Even though plasma glucose and HbA1c are not directly comparable, this finding supports the results of Ziegler et al.^11^, who found an increasing prevalence of CAN over categories of increasingly abnormal glucose levels. Jarczok et al.^25^ presented a scatterplot of fasting glucose against the frequency domain metric HF which looks visually almost identical to our Fig. 3A. They used locally weighted regression (lowess) to fit regression lines, which were nearly linear as well (in the normal glucose range), and in agreement with our results for the GAMs.

According to hypothesis 3, we investigated whether the association between ACR and PRV would hold regardless of whether HbA1c was included in the model or not, and the primary interest was not in an interaction term between the two. We found that the assumption underlying the hypothesis, that ACR was associated with PRV at all, was weak. This relationship was only significant in the models that used SDNN as outcome. Furthermore, statistical significance for ACR (log transformed) vs PRV remained only for one of the two SDNN outcomes after inclusion of HbA1c, and we are therefore unable to conclude whether HRV is associated with ACR independently of HbA1c based on our data.

In three of the four models, we found a statistically significant interaction between log ACR and HbA1c, in which there was a negative association between log ACR and HRV for low values of HbA1c, but a positive association for higher values of HbA1c. In the model with post-CPT SDNN as outcome and only age and sex as covariates (without HbA1c), the coefficient for log ACR was − 2.9. This number means that if the ACR is doubled, the expected decrease in SDNN is 0.9 ms. The corresponding number in the pre-CPT model was 0.4 ms per doubling.

For participants with albuminuria, there was no visible trend between SDNN and HbA1c (supplementary figure S4), possibly due to a low sample size for this group. For the participants without albuminuria, the SDNN-HbA1c trend was negative, in accordance with the overall association shown in Fig. 3B.

When the main analysis is done separately in the CVD and healthy groups, we see that the confidence intervals for the CVD group are much wider, making it hard to detect any pattern of PRV over MetS levels for the participants with CVD. One obvious reason for the wider intervals is that there are much fewer participants with known CVD than without, as the interval width decreases with sample size.

However, it is also possible that the CVD group has a higher natural variance because different types of CVD were included in this analysis, ranging from angina without damage to small troponin infarctions to large infarctions with heart failure. As we do not see a big difference between the estimated standard deviations for the CVD and healthy group, the wide confidence intervals are more likely due to the relatively low number of subjects with CVD.

This study has some limitations. First, the Tromsø 6 sample does not correspond to the age distribution of the general population, but is skewed towards older participants, in accordance with the Tromsø 6 inclusion strategy. Second, we did not have access to fasting glucose for determining the MetS glucose criteria. This could potentially lead to bias if healthy participants were misclassified as fulfilling the glucose criteria because they had elevated glucose levels following a meal. However, this limitation does not seem to have affected the results notably, as the glucose component contributed significantly to all four HRV outcomes (Table 4), and there were clear differences between the healthy controls and MetS groups in most analyses. Initially, we attempted to incorporate the self-reported time since the last meal variable in the definition, but that likely caused some participants with elevated glucose to be misclassified as controls. The criterion was consequently defined without that variable. This process is explained in detail in the supplementary information.

Third, some of the variables in the study are fully or partially based on self-report, such as diabetes and age at diagnosis, time since last meal, and CVD information that was not registered in the end-point registry. Self-reported data can be affected by biases, such as recall bias or social desirability bias. However, since only the age at diabetes diagnosis goes far back in time, and the questions are not particularly sensitive, any bias from self-reporting for these variables is expected to be modest.

Tromsø 6 has a good gender balance and a high response rate. It also has the advantage of having a large number of participants, giving enough statistical power to answer detailed questions in the main hypothesis. Still, when looking into subgroups such as those with albuminuria or known CVD, it is possible that the lack of detected associations is due to an insufficient statistical power.

In this study we have used ultra-short-term PRV (30 or 60 s) based on plethysmographic measurement as a surrogate for HRV that is conventionally derived from longer recordings of ECG. As it is known that PRV may deviate from ECG-based HRV^15^ and that ultra-short-term HRV is not completely validated as a surrogate^17^, our PRV data possibly contains noise components of either technical or physiological origin that increase variation and could have reduced the statistical power of the dataset. In order to reduce noise in the dataset, the data had previously been cleaned for artifacts using automated procedures^33^ and recordings with the highest PRV (most likely to contain artifacts) were visually inspected in this study and excluded if errors were suspected. Some studies have found ultra-short-term HRV to be good surrogates (for five-minute recordings) depending on the recording length and the type of HRV metric. With respect to the metrics used in this study (RMSSD and SDNN), Baek et al.^18^ found that 30 s and 240 s was required to reliably estimate 5-min RMSSD and SDNN respectively. Shaffer et al.^34^ found that resting baselines as brief as 1 min should be sufficient to measure SDNN and RMSSD in their study population. Munoz et al.^20^ confirmed that it is unnecessary to use recordings longer than 120 s to obtain accurate measures of RMSSD and SDNN. With respect to diabetes patients, Nussinovitch et al.^35^ found that ultra-short (10 s) RMSSD and SDNN (1 min) were reliable markers for 5-min HRV. In our study, PRV was derived from 30 s (pre-CPT) and 60 s (post-CPT) recordings. Although RMSSD seems to require shorter recordings, this metric also seems to be more sensitive to recording artifacts from photoplethysmography-based PRV compared to SDNN^16^. Thus, the accuracy of our SDNN data may be reduced by the short recording length, while the RMSSD accuracy is more likely reduced as a consequence of plethysmography-based measurement.

The results of this study are of interest for applications based on PRV measurement for the purpose of predicting risk of complications associated with metabolic syndrome such as cardiovascular disease. The technology behind PRV measurement is simple to miniaturize and is already available in smart-watches^36,37^. It is important to note that although there is a significant difference on a population level, this does not guarantee that the data has the precision needed for a clinically useful test. The ability to predict outcomes of interest based on this short test must be investigated in a further study. PRV may also be valuable as an addition to the metabolic syndrome criteria for risk prediction at the individual level, but this needs to be confirmed by further studies. The large variance in the PRV data also indicates that experimental or technical factors could have influenced the measurement and that the reproducibility may be low. For predictions at the individual level, the measurement reproducibility must be optimized by consideration of the testing situation, measurement instrumentation and the signal processing used to derive PRV variables. More reliable PRV data may be acquired by, for instance, smart watches, during sleep and other contexts where individual changes can be tracked over time.

Having metabolic syndrome is defined as meeting at least three of the five criteria^3^. This definition corresponds well with our findings on PRV from the Tromsø 6 population, where the PRV drops from none to three MetS levels, but plateaus at the lowest level from three levels and above (Fig. 2). The PRV had significantly decreased already at the first MetS level, indicating that changes in cardiac or autonomous regulation may be detected at the earliest stages of MetS in individuals considered healthy. This could be important, as symptoms of autonomic deficits often appear late in CAN, when reversibility is limited. Screening for CAN at an early stage could help manage or reverse the progression^10^.

The MetS and DM populations are different from healthy controls with respect to PRV, indicating an impaired autonomic nervous system in both conditions, but we could not find a stronger alteration in the DM versus the MetS population in our sample. This study supports the notion that both the MetS components and manifest diabetes affect the autonomic nervous system in this population. In the future, a study with assessment of PRV measurements in relation to prospective cardiovascular events seems justified.

## Methods

### Data source

The Tromsø Study is a prospective epidemiological study of health problems, symptoms, and chronic diseases initiated in 1974. So far, 7 surveys have been carried out. This study is based on data from the sixth wave, Tromsø 6, which was performed in 2007–2008; 19,762 participants aged 30–87 and of both genders were invited and 12,984 (65.7%) participated in the first of two visits (53% women). Participants were invited from four groups: All participants from the second visit of the fourth Tromsø Study taking place in 1994–1995, and in addition a 10% random selection of inhabitants between 30 and 39 years, all inhabitants between 40–42 years and 60–87 years, and a 40% random selection of inhabitants between 43 and 59 years. The age distribution was similar for both sexes.

The Tromsø study maintains a cardiovascular end-point registry that contains verified CVD diagnoses. This was used in addition to questionnaire data from Tromsø 6 to get information about the participants’ history of CVD.

The Norwegian Data Protection Authority and the Regional Committee of Medical and Health Research Ethics, North Norway have approved the Tromsø 6 study. The study complies with the Declaration of Helsinki, and each participant gave written informed consent prior to participation. This study is a part of a project called “Diabetes and hemodynamics: a big data study with statistical modeling and machine learning methods in the sixth health survey of the Tromsø Study,” that was approved by the Research Ethical Committee of Northern Norway (2019/143).

### Measurements and data collection

**Figure 5:**
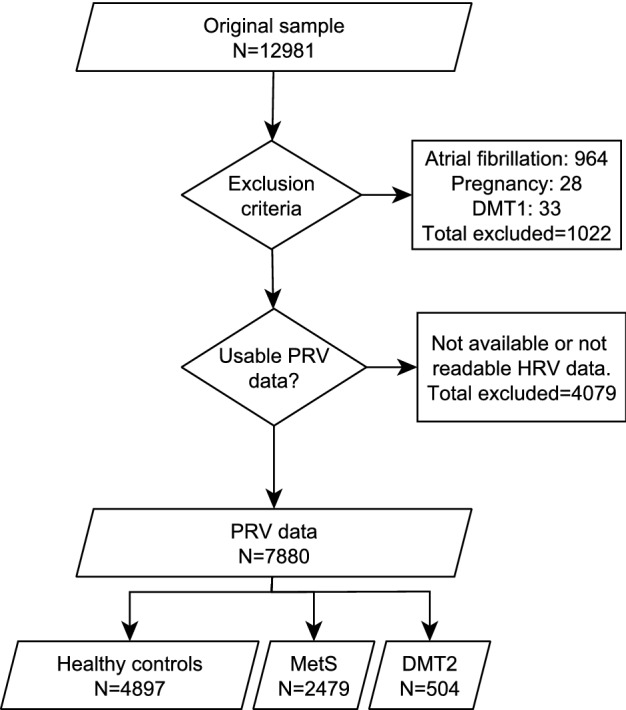
Flowchart describing the sample of HRV data for healthy controls, subjects with metabolic syndrome and diabetes type 2 derived from the Tromsø 6 study. DMT1 diabetes mellitus type 1, DMT2 diabetes mellitus type 2, MetS metabolic syndrome.

Tromsø 6 included several questionnaires and interviews with questions on a wide variety of demographic, social and health-related topics, anthropometric measurements, clinical examinations and the sampling of blood and other biological materials. Sampling procedures can be found in^38^. In addition, there were data from 10,584 participants who underwent a cold pressor test (CPT)^39–41^ to evaluate pain sensitivity and cardiovascular reactivity. They were continuously monitored with a non-invasive beat-to-beat blood pressure monitor (Finometer Pro; Finapres Medical Systems, Amsterdam, The Netherlands) before, during and after the test. The data from the Finometer Pro was used to calculate estimates of HRV as described by Bruehl et al.^33^. Briefly, short recordings of inter-beat intervals before (approximately 30 s) and after the CPT (approximately 60 s) were cleaned for artifacts and used to calculate different HRV parameters using the RHRV package in R. The recordings during the test were not used in this study. After exclusion of pregnant women, recordings with technical errors and participants with diabetes mellitus type 1 (DMT1) or atrial fibrillation, there were 7880 subjects with readable HRV measurements that were included in the study (Fig. 5). A subgroup of the original sample (N = 7,306) attended a second visit in Tromsø 6 that included sampling of biological specimens and clinical examinations. Three urine specimens from 7,196 subjects were collected.

### Variable selection

In order to answer the research questions, the variables presented in Table 2 were selected for analysis. From these, additional variables were derived, such as the components of the metabolic syndrome.

### Data processing

Among the selected predictor variables, there was a substantial number of missing values (10% of the variable matrix of participants who attended the CPT) and excluding all participants with any missing data points would leave only 46% for further analysis. Missing values were therefore imputed employing the multivariate multiple imputation method^42^ using the MICE package in R^43^ with the default imputation models for continuous and categorical variables. Derived variables (such as the MetS components), were not included in the imputations, following the *impute, then transform* approach^44^. As not all missing values needed imputation (planned missing, e.g., not attending the second day), the *where-matrix* in the mice function was manipulated accordingly. Variables with planned missing were not allowed to predict other variables (controlled through the *predictorMatrix* argument in the mice function) as this would make the missing values propagate through the dataset. However, the variables from day 2 were allowed to predict each other. To supply the imputation model with more data, rows with missing outcome variables were also included. The missing outcome was imputed (to not let them propagate), but all rows with imputed outcome variables were removed before the analysis. The dataset was imputed 30 times, analyzed and pooled to include the uncertainty from the imputation process in the estimates. The imputation was done separately for each hypothesis. We assumed that the missing data was missing at random.

Some participants had abnormally high SDNN or RMSSD measures that were regarded as outliers. In order to determine whether or not to include these in the analysis, visual inspection of the inter-beat intervals was done for all SDNN values at or above 150, both pre-CPT (106 recordings) and post-CPT (221 recordings). This resulted in the exclusion of 32 and 59 measurements from each time period respectively, based on likely technical measurement errors. The participants were not removed from the dataset, but the HRV measure from the relevant time period was deleted.

Participants were classified as having diabetes type 2 if they had HbA1c at 48 mmol/mol or greater, used glucose lowering medications or if they reported having diabetes in the questionnaire. Participants were excluded as having diabetes type 1 if they reported being diagnosed before or at the age of 30. We used the harmonized MetS criteria^3^, which stated that metabolic syndrome should be diagnosed if three or more of the following five criteria were met:

1. Waist circumference ≥ 94 cm (men)/ ≥ 80 cm (women) (Europids)
2. Triglycerides ≥ 1.7 mmol/l
3. High density lipoprotein (HDL) cholesterol < 1.0 mmol/l (men)/ < 1.3 mmol/l (women)
4. Blood pressure ≥ 130 mm Hg systolic and/or ≥ 85 mm Hg diastolic
5. Fasting glucose ≥ 5.6 mmol/l

We also considered criteria 2, 3, and 4 to be met if the participant was on drug treatment for the condition at the time. The glucose criterion in the definition of MetS reflects fasting glucose, but the participants in this study were not asked to fast before attendance. We did not use the self-reported time since last meal variable in the definition of the criterion, the justification for this can be found in the supplementary information. Instead, the criterion was defined as follows:

- Diabetes: HbA1c ≥ 48 mmol/l OR use of medications OR self-report
- Glucose criterion: Glucose ≥ 5.6 mmol/l AND do not have diabetes
- Healthy control: None of the above

CVD was defined as myocardial infarction, angina and stroke. For myocardial infarction and stroke, information from the cardiovascular end-point registry was combined with the information from the questionnaires. As angina is not in the registry it is based on the questionnaire alone. Albuminuria was measured as the albumin-creatinine ratio (ACR) calculated as the average ratio from three different measurements. This was only available from the subgroup of participants that attended the second visit, and 4426 participants had both ACR data and readable PRV data.

### Statistical testing

Using the Tromsø 6 dataset, our aim was to investigate how the metabolic syndrome and diabetes type 2 is associated with changes in HRV. Our primary hypothesis was that altered HRV is related to the number of metabolic syndrome components, with the strongest alteration in manifest diabetes. To answer this, a variable counting the number of fulfilled MetS criteria per individual was constructed, with a separate category for participants with diabetes (independent of their number of MetS criteria). This will from now on be referred to as the MetS levels. Not assuming a linear relationship, this variable was used as a categorical independent variable in an ANCOVA model. To further investigate differences between specific groups, contrast analysis and Tukey tests were used.

In order to estimate how each specific MetS component and diabetes contributed to alterations in PRV, a model with the MetS components (as binary variables) and diabetes was employed. Main effects and interactions up to the three-way level were assessed.

In addition, we aimed to test three secondary hypotheses:

1. Alteration in HRV increases with increasing HbA1c level.
2. HRV is associated with albuminuria independently of HbA1c
3. Alteration in HRV increases with increasing number of metabolic syndrome components, with the strongest alteration in manifest diabetes in subjects with and without known CVD.

Linear regression and generalized additive models (GAM) were used to explore the relationship between PRV and HbA1c. As the GAM package (mgcv) was not compatible with imputation, results from unimputed data are presented. To test whether the relationship between PRV and albuminuria is independent of the level of HbA1c, linear regression with log transformed ACR was used. To answer the last hypothesis, the analysis of the main hypothesis was repeated with data grouped on whether the participants had known CVD or not. The analyses were repeated with SDNN and RMSSD, both before and after the CPT, as outcome variables. All models were adjusted for sex and age (centered) and all statistical tests were two-sided and done with a fixed significance of 0.05. All analyses were done in R (version 4.0.3).

## Supplementary Information

Supplementary Information.

## Data Availability

The dataset analyzed in the current study is not publicly available, but access can be requested from the Tromsø study (https://uit.no/research/tromsostudy/project?pid=709148). To respect terms of use and protect human privacy, the authors cannot directly share the dataset.

## Code availability

The code used for statistical analysis is available from the authors on request.
