# Supplementary material for: Reduced heart rate variability is related to the number of metabolic syndrome components and manifest diabetes in the sixth Tromsø study 2007–2008
Source: Sci Rep. 2022 Jul 14;12:11998. doi: 10.1038/s41598-022-15824-0 (PMC9283528; doi:10.1038/s41598-022-15824-0)
Supplement: Supplementary file 1 — Supplementary Information. [file 41598_2022_15824_MOESM1_ESM.pdf]

Supplementary Information

Reduced heart rate variability is related to the number of metabolic syndrome components and manifest diabetes in the sixth Tromsø study 2007-2008

Naomi Azulay, Roy Bjørkholt Olsen, Christopher Sivert Nielsen, Audun Stubhaug, Trond Geir Jenssen, Henrik Schirmer, Arnoldo Frigessi, Leiv Arne Rosseland, Christian Tronstad

Since the participants had not been fasting before the blood test, but there was information on the time since their last meal (TSLM), we initially defined the glucose criterion with a condition of fasting for at least 4 hours prior to blood sampling, or else they were put in the control group. This led to a mixing of the groups that had elevated glucose due to meals and the participants with elevated fasting glucose level. When comparing mean HRV between three groups with increasing glucose (defined by thresholds 5.6 and 7 mmol/l), there was a clear negative trend in HRV. At the same time, SDNN was significantly lower among those with TSLM <4 hours (non-fasting) compared to TSLM ≥ 4 hours (fasting). This might be a result of the meal, as Sauder et al.[1] found reduced HRV (HF) values 2 hours after a meal compared to after 6 hours fasting. Accepting non-fasting participants as part of the control group regardless of their glucose levels altered the proportion of non-fasting to fasting, and lead to a higher mean SDNN in the glucose group than the control group (table S1). With this in mind, and considering Moebus et al.[2] did not find big differences in fasting and non-fasting glucose levels, we chose to not adjust the glucose criteria based on TSLM.

Table S1. Mean SDNN pre-CPT in the diabetes, glucose, and control groups when the glucose criterion was defined with and without a condition of time since last meal (TSLM) ≥ 4.

	Definition with TSLM	Definition without TSLM
Control	39.7	40.3
Glucose criterion	40.3	36.3
Diabetes	32.3	32.3

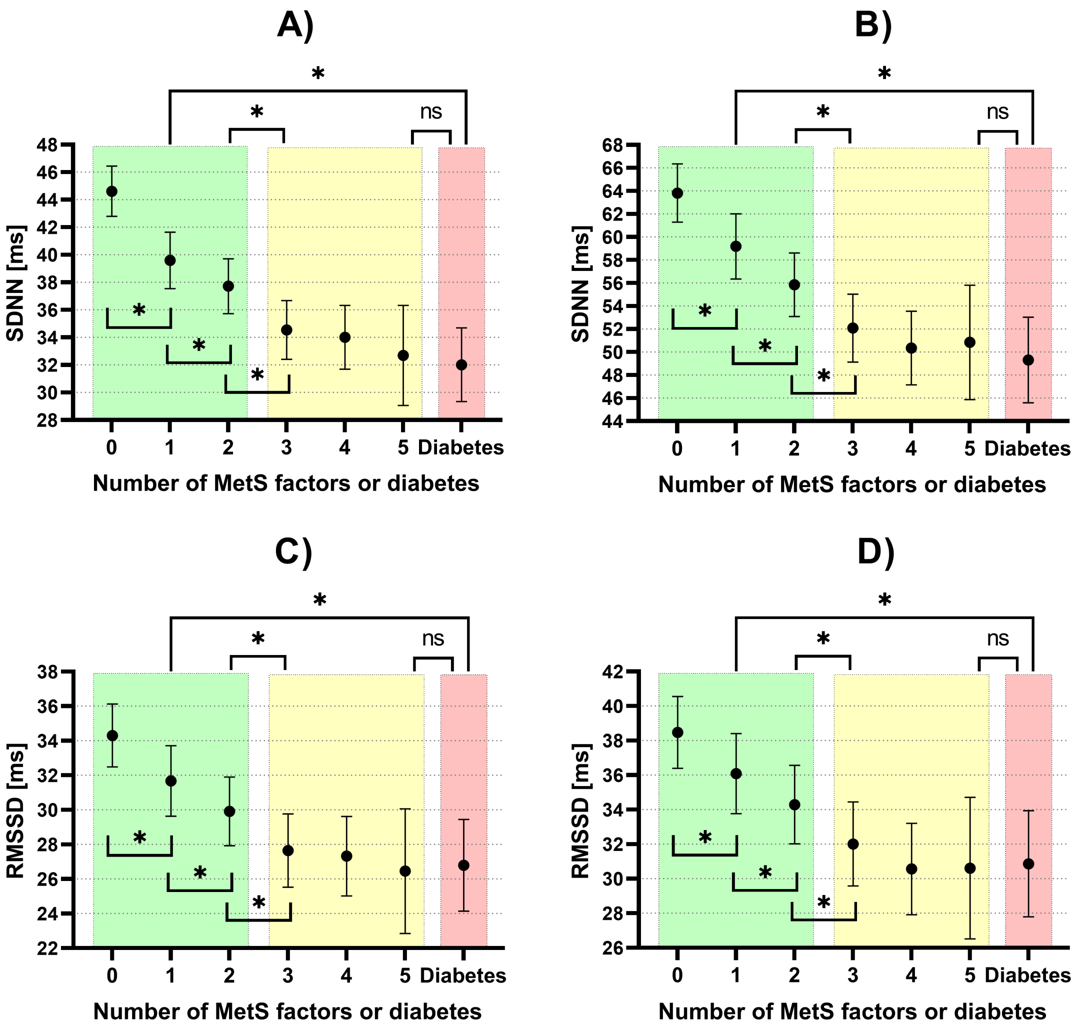

*Figure S1. Mean and 95 % confidence intervals of PRV for participants having different numbers of metabolic syndrome components or diabetes presented for pre-CPT SDNN (A), post-CPT SDNN (B), pre-CPT RMSSD (C) and post-CPT RMSSD (D). Green area represents healthy subjects and yellow area represents subjects with metabolic syndrome according to current definitions. *p<0.05 from contrast analysis and Tukey's tests. Only Tukey tests from adjacent groups are shown in this plot, for a complete overview, see figure S2.*

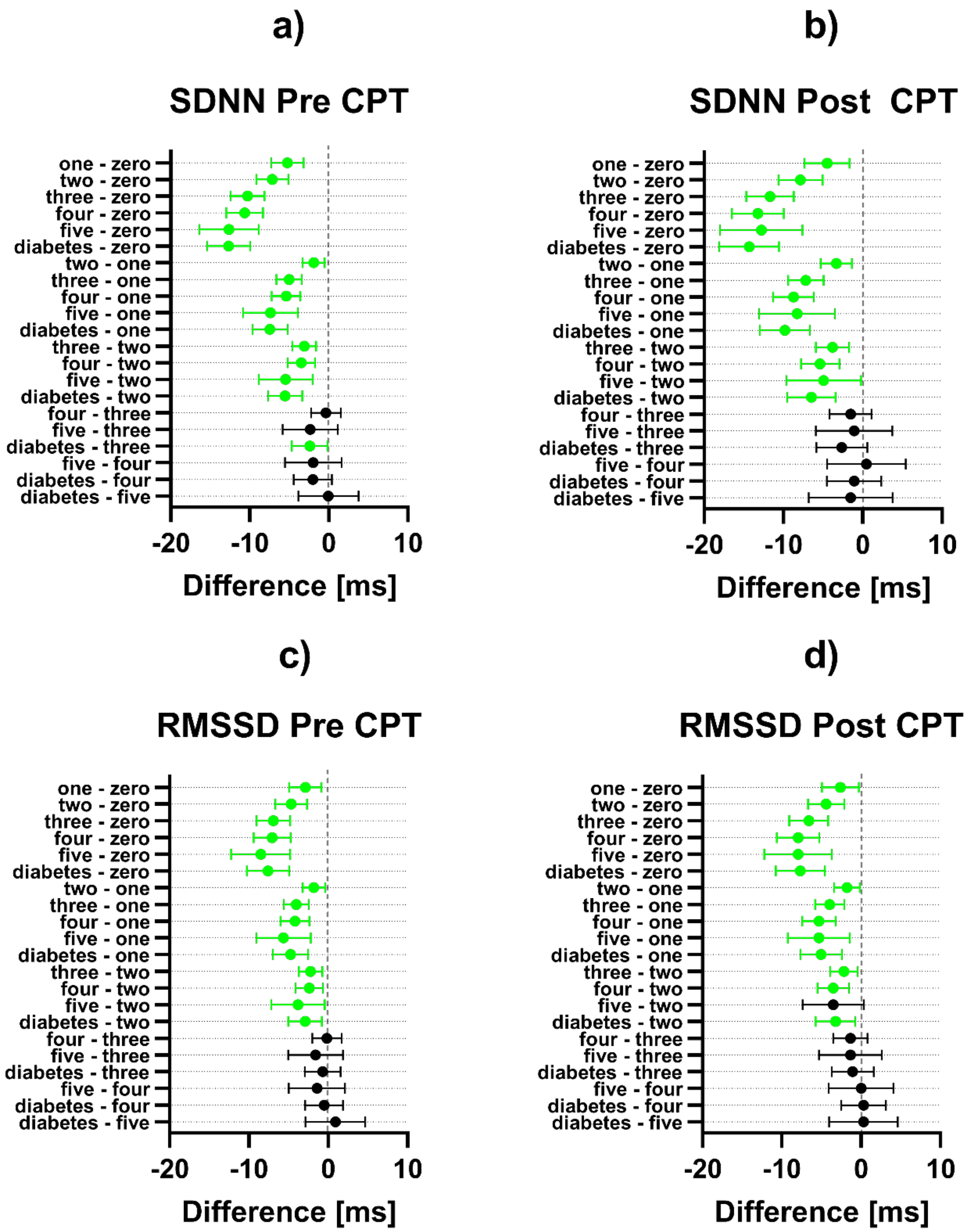

Figure S2. Estimates of and 95 % confidence intervals for pairwise differences between all metabolic syndrome levels and diabetes based on Tukey's method. Green color indicates a significant difference.

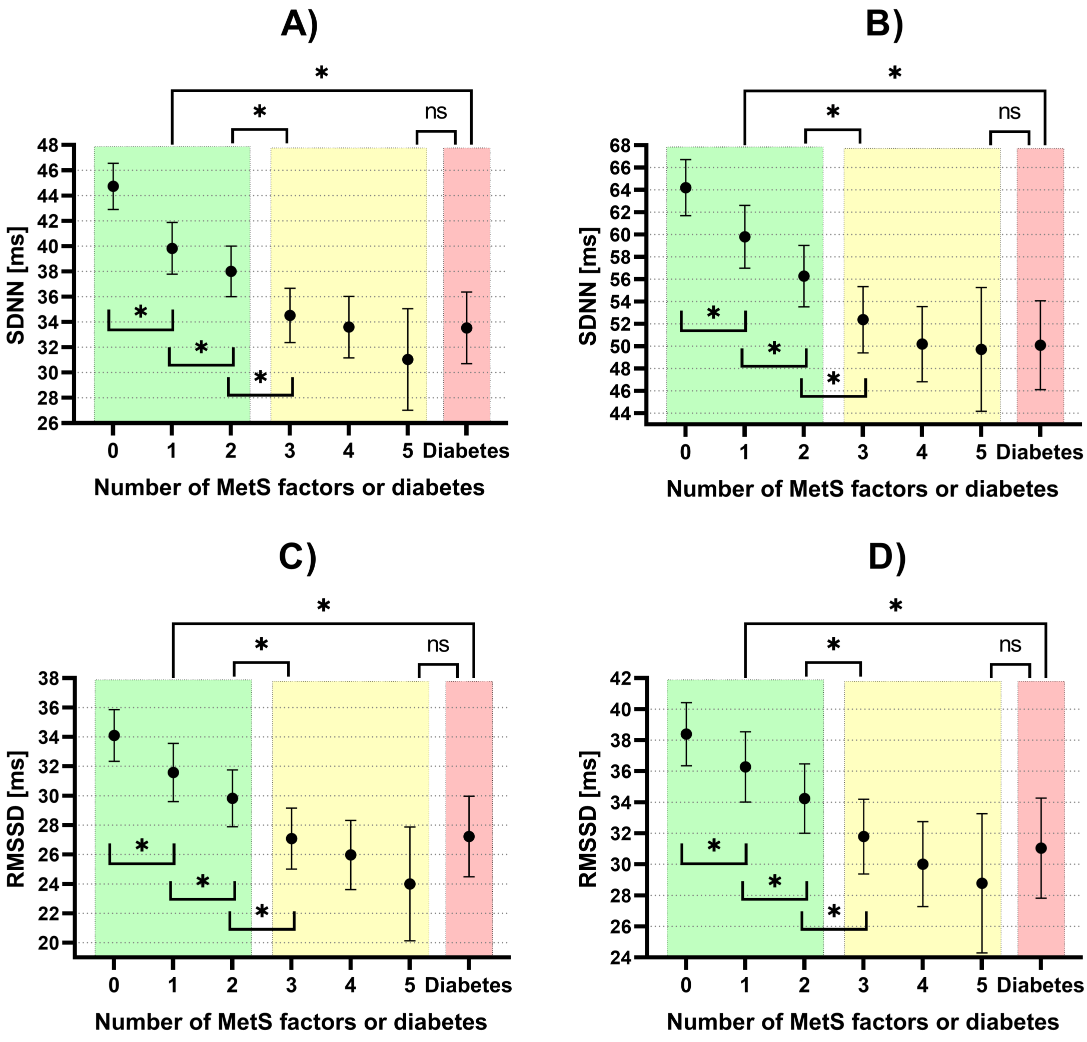

*Figure S3. Analysis (same as Figure S1) excluding participants on beta blockers: Mean and 95 % confidence intervals of PRV for participants having different numbers of metabolic syndrome components or diabetes presented for pre-CPT SDNN (A), post-CPT SDNN (B), pre-CPT RMSSD (C) and post-CPT RMSSD (D). Green area represents healthy subjects and yellow area represents subjects with metabolic syndrome according to current definitions. There were 650 participants on beta blockers (626 post CPT). *p<0.05 from contrast analysis and Tukey's tests. Only Tukey tests from adjacent groups are shown in this plot.*

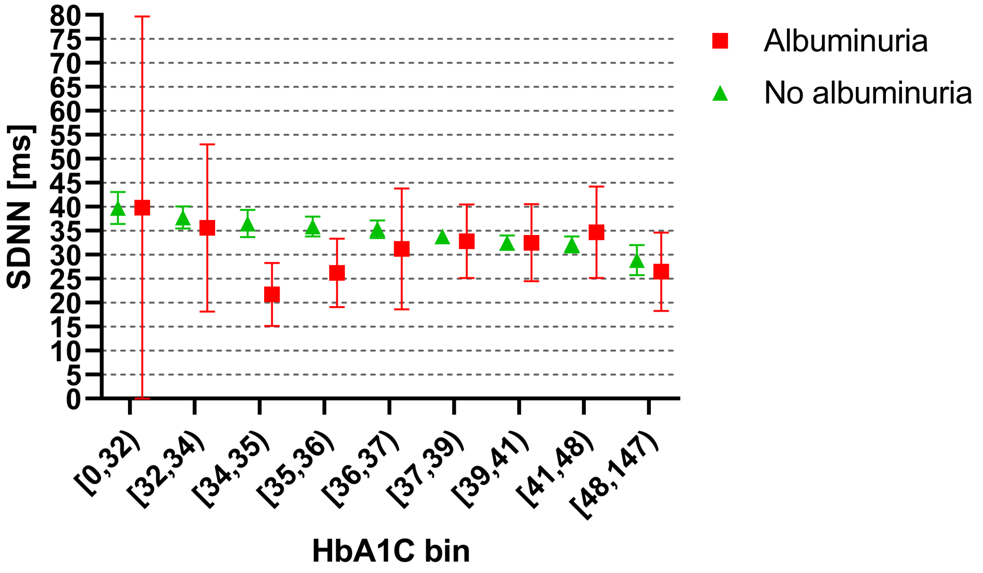

Figure S4. Mean and 95 % confidence interval of SDNN pre-CPT for participants grouped according to different HbA1c ranges and stratified on albuminuria (moderately or severely increased) status. 281 participants had albuminuria.

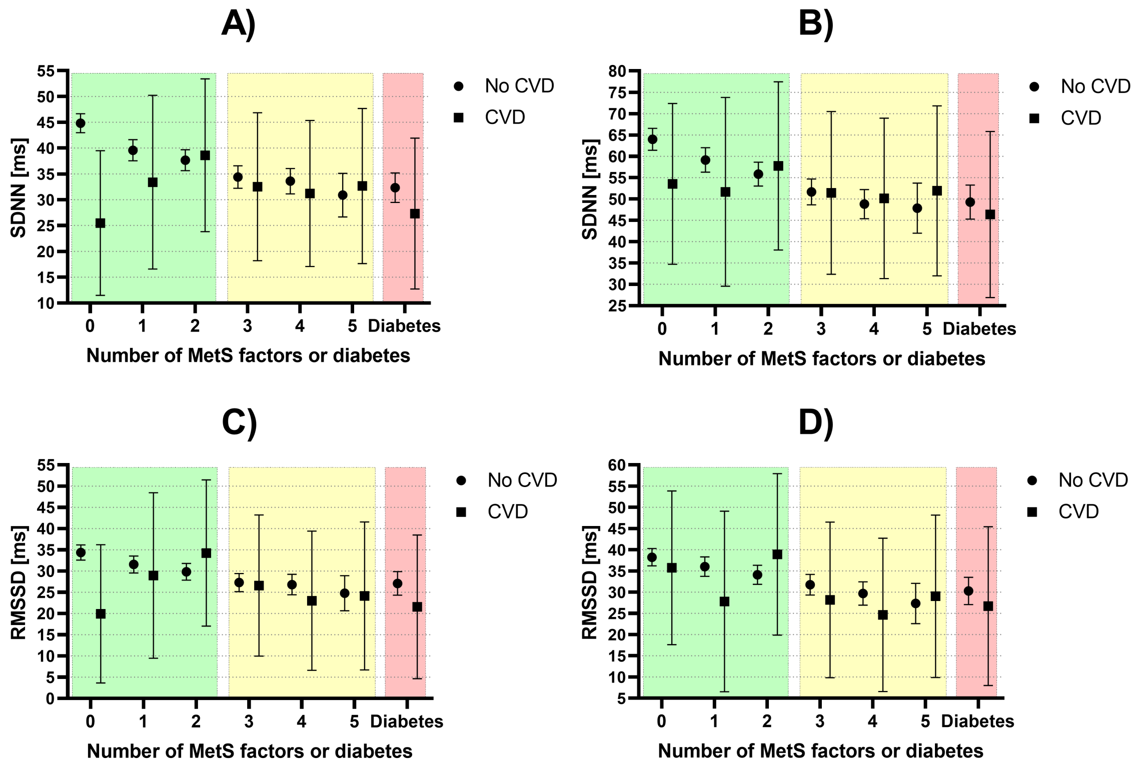

Figure S5. Mean and 95 % confidence intervals of PRV for participants having different numbers of metabolic syndrome components or diabetes, stratified on CVD status presented for pre-CPT SDNN (a), post-CPT SDNN (b), pre-CPT RMSSD (c) and post-CPT RMSSD (d). Green area represents healthy subjects and yellow area

represents subjects with metabolic syndrome according to current definitions. Only 636 participants had known CVD (607 post CPT), resulting in wider confidence intervals compared to the group without CVD.

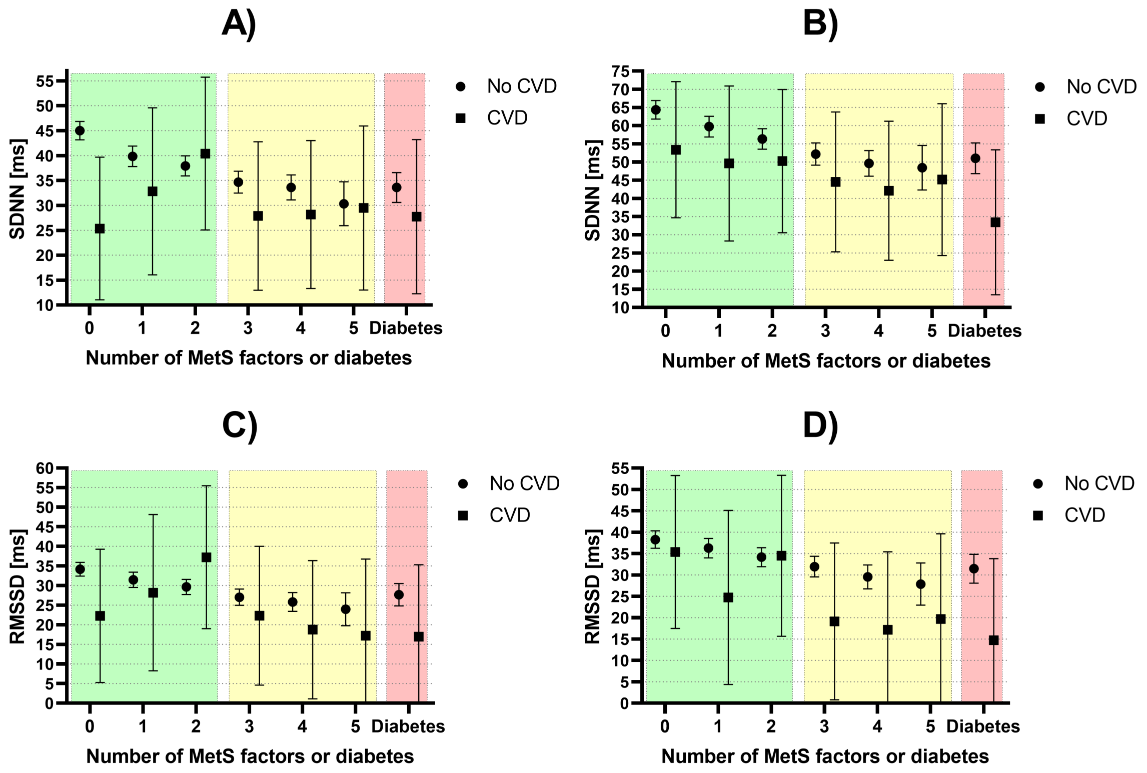

Figure S6. Analysis (same as Figure S5) excluding participants on beta blockers: Mean and 95 % confidence intervals of PRV for participants having different numbers of metabolic syndrome components or diabetes, stratified on CVD status presented for pre-CPT SDNN (a), post-CPT SDNN (b), pre-CPT RMSSD (c) and post-CPT RMSSD (d). Green area represents healthy subjects and yellow area represents subjects with metabolic syndrome according to current definitions. Only 296 participants that were not on beta blockers had known CVD (280 post CPT), resulting in wider confidence intervals compared to the group without CVD.

References

1. Sauder, K. A., Johnston, E. R., Skulas-Ray, A. C., Campbell, T. S. & West, S. G. Effect of meal content on heart rate variability and cardiovascular reactivity to mental stress. *Psychophysiology* **49**, 470–477 (2012).
2. Moebus, S., Göres, L., Lösch, C. & Jöckel, K. H. Impact of time since last caloric intake on blood glucose levels. *European Journal of Epidemiology* **26**, 719–728 (2011).
